# Relationships between Nutrient-Related Plant Traits and Combinations of Soil N and P Fertility Measures

**DOI:** 10.1371/journal.pone.0083735

**Published:** 2013-12-31

**Authors:** Yuki Fujita, Peter M. van Bodegom, Jan-Philip M. Witte

**Affiliations:** 1 Team Ecohydrology, KWR Watercycle Research Institute, Nieuwegein, The Netherlands; 2 Department of Systems Ecology, VU University Amsterdam, Amsterdam, The Netherlands; DOE Pacific Northwest National Laboratory, United States of America

## Abstract

Soil fertility and nutrient-related plant functional traits are in general only moderately related, hindering the progress in trait-based prediction models of vegetation patterns. Although the relationships may have been obscured by suboptimal choices in how soil fertility is expressed, there has never been a systematic investigation into the suitability of fertility measures. This study, therefore, examined the effect of different soil fertility measures on the strength of fertility–trait relationships in 134 natural plant communities. In particular, for eight plot-mean traits we examined (1) whether different elements (N or P) have contrasting or shared influences, (2) which timescale of fertility measures (e.g. mineralization rates for one or five years) has better predictive power, and (3) if integrated fertility measures explain trait variation better than individual fertility measures. Soil N and P had large mutual effects on leaf nutrient concentrations, whereas they had element-specific effects on traits related to species composition (e.g. Grime's CSR strategy). The timescale of fertility measures only had a minor impact on fertility–trait relationships. Two integrated fertility measures (one reflecting overall fertility, another relative availability of soil N and P) were related significantly to most plant traits, but were not better in explaining trait variation than individual fertility measures. Using all fertility measures together, between-site variations of plant traits were explained only moderately for some traits (e.g. 33% for leaf N concentrations) but largely for others (e.g. 66% for whole-canopy P concentration). The moderate relationships were probably due to complex regulation mechanisms of fertility on traits, rather than to a wrong choice of fertility measures. We identified both mutual (i.e. shared) and divergent (i.e. element-specific and stoichiometric) effects of soil N and P on traits, implying the importance of explicitly considering the roles of different elements to properly interpret fertility–trait relationships.

## Introduction

Soil nutrients, such as nitrogen (N) and phosphorus (P), constitute basic requirements for plants to support their life. In the short term, soil nutrients induce plastic responses of individual plants (e.g. as related to recruitment, growth, and reproduction). In the longer run, soil nutrients affect the species composition of a plant community, because each species has evolved to adapt to certain environments and therefore has contrasting requirements for nutrients. How and why plant community composition changes from fertile to unfertile soil has been a central concern of ecologists for many decades. A general trend derived from qualitative studies is that species with a rapid growth strategy dominate in fertile soils, whereas species with a conservative strategy dominate in infertile soils (for a review see [1]). In quantitative trait-based studies on a global scale, between-site variations of leaf traits were well explained by soil fertility if combined with climate factors (R^2^ between 59 and 78% for specific leaf area and leaf N and P concentrations) [2]. Within a climatic region, however, the effects of soil fertility on leaf traits were moderate (e.g. 22–23% of between-site variance in an integrated leaf economy measure were explained by nutrient indicator value [3]; 31% and 50% of between-site variance in leaf N and P concentrations were explained by soil total P if combined with growth form [4]). Accordingly, the relatively weak relationship between soil fertility and plant traits remains an uncertain link in trait-based species distribution models [5].

Generally weak linkages between environmental factors and traits could be due to stochastic processes being more dominant than environmental filtering effects during the assembly of these plant communities [6], [7]. However, these relatively weak relationships could also be the results of using suboptimal (or inappropriate) measure of soil fertility [3]. Soil fertility, or availability of nutrients for plants, is typically expressed as concentrations of dissolved N or P in soil, N mineralization rates for a certain period, soil total N or P, or soil C:N or C:P ratios. Although these soil fertility measures are generally correlated [2], [8], it is likely that the choice of a fertility measure influences the strength of the correlation between soil fertility and plant traits (e.g. [9]). Nevertheless, whether the most relevant fertility measure was used to examine the relation with a specific plant trait has never been explicitly examined. For example, the two nutrient elements, N and P, both have pivotal yet different roles in how plants function. Previous studies showed that soil N and P have different degrees of contributions to each trait: specific leaf area was better predicted by soil N supply, whereas leaf N and P concentrations showed stronger relationships with soil P [2], [4]. This implies that element-specific mechanisms or stoichiometric effects (i.e. effects of relative availability of N and P) may play important roles in regulating these traits.

Furthermore, any measure of soil fertility reflects the nutrient status over a certain timescale. This timescale is implicitly (e.g. total N) or explicitly (e.g. N mineralization over 5 years) reflected in the way the measure is expressed. Measures from chemical soil bulk analysis at a certain moment, such as pool size and extractable amount of nutrients, provide only partial information about the nutrient availability for plants over a long time span [10]. Nutrient mineralization rates from soil could be a more relevant measure of nutrient availability for living plants as they reflect a major flow within soil nutrient cycles [11]. However, they fluctuate considerably over time and are controlled by abiotic factors such as temperature and soil moisture [12]. Thus, the timescale associated with soil fertility measures could influence the relationship between soil fertility and plant traits.

Moreover, since plants use soil nutrients in a multidimensional manner (e.g. different ratios of elements, at different timings and periods, etc.), certain combinations of soil fertility measures may better reflect plant trait variability. Hence, an aggregated measure derived from multiple fertility measures may better explain trait variability than a single fertility measure.

This study aims to investigate whether the relationships between soil fertility and nutrient-related plant traits can be improved by analyzing the mutual and divergent impacts of different soil fertility measures. To this aim, we tested (1) whether nutrient elements in soil (N or P) have specific or shared impacts on plot-mean plant traits (i.e. averaged or aggregated values of traits of all plants in each plot), (2) whether the timescale of a soil fertility measure is important in explaining plot-mean plant traits, and (3) whether use of an integrated soil fertility measure improves the fertility vs. trait relationships. Subsequently, we infer from our findings which fertility measure or combinations of fertility measures are the best suited to describe among-plots trait variations. Throughout the paper, we considered vascular plants only, since non-vascular species (i.e. mosses and lichens) do not directly take up nutrients from soils and are thus less relevant in the context of soil fertility–trait relationships.

## Materials and Methods

### Ethic Statement

We obtained permissions for soil and vegetation sampling in Hoge Veluwe national park (permission from Stichting het nationale park de Hoge Veluwe), Zuid-Kennemerland national park (permission from Provinciaal Waterleidingbedrijf Noord-Holland), and nature reserves owned by Staatsbosbeheer.

### Site selection

We selected 36 sites in the Hoge Veluwe and Zuid-Kennemerland national parks and nature reserves owned by Staatsbosbeheer (dataset 1). Additionally, we used 47 sites of Olde Venterink et al. [11] (dataset 2) and 51 sites of Ordonez et al. [3] (dataset 3); these studies used almost the same methods to measure soil and plant properties (see Table 1 for a methodological overview). In total, we thus acquired information for 134 sites and 372 plant species in natural ecosystems in the Netherlands and Belgium, consisting of 104 grasslands, 17 shrub lands (including heath), and 13 forests. These sites cover the range of ecosystems typical in this region except for those influenced by brackish water. Vascular plant species were recorded in a plot size of 4 m^2^ for herbaceous, 4, 9, or 25 m^2^ for shrub (depending on the size of the woody species), and 100 m^2^ for forest stands. Soil cores of 10 cm depth (datasets 1 and 3) or 15 cm depth (dataset 2) were taken within or next to the plot. Large roots were removed from the soil cores. For datasets 1 and 3, several soil cores were mixed to make a composite soil to eliminate the effects of local soil heterogeneity. In the peak growing season (July or August), above-ground standing biomass of vascular plants (datasets 1 and 2) or leaves of dominant vascular species (dataset 3) were harvested.

**Table 1:** Overview of methodology of soil and plant trait measurements for three datasets used in this study.

		Dataset 1	Dataset 2 [11]	Dataset 3 [3]
N. of sites		36	47	51
N. of combined soil cores per site		3	1	5
*Soil fertility measurements*				
Soil C	%	CNS analyzer^*1*2^	0.5 · Loss on ignition at 550°C	CNS analyzer*1
Soil N	%	CNS analyzer*1	Kjeldahl digestion	CNS analyzer*1
Soil P	%	HNO_3_ + HCl digestion	Kjeldahl digestion	HNO_3_ + HCl digestion
Dissolved N (N–NO_3_ + N–NH_4_)	mg N kg^−1^ dry soil	1 M KCl extraction	1 M KCl extraction	1 M KCl extraction
Dissolved P (P–PO_4_)	mg P kg^−1^ dry soil	Olsen extraction (0.5 M NaHCO_3_)	ALA extraction (0.1 M NH_4_OH + 0.1 M lactic acid + 0.4 M acetic acid)	Olsen extraction (0.5 M NaHCO_3_)
Summer N mineralization	mg N kg^−1^ dry soil week^−1^	6 weeks in-situ incubation in May–July (d 15 cm x ø 4 cm)	8 weeks in-situ incubation in July–August (d 10 cm x ø 4.8 cm)	6 weeks in-situ incubation in June–August (d 15 cm x ø 6 cm)
Annual N and P mineralization	mg N (or mg P) kg^−1^ dry soil 243-day^−1^	Simulated with CENTURY	Simulated with CENTURY	Simulated with CENTURY
5-year N and P mineralization	mg N (or mg P) kg^−1^ dry soil 5-year^−1^	Simulated with CENTURY	Simulated with CENTURY	Simulated with CENTURY
Soil texture*3	clay/silt/sand in fraction	Laser particle sizer	Estimated*4	Estimated*4
*Plant trait measurements*				
LNC	mg g^−1^	-	-	CNS analyzer*1
LPC	mg g^−1^	-	-	HNO_3_ + HCl digestion
WNC	mg g^−1^	CNS analyzer*1	Kjeldahl digestion	-
WPC	mg g^−1^	HNO_3_ + HCl digestion	Kjeldahl digestion	-
IV_nut_	-	Derived from species composition		
CSR	-	Derived from species composition		

^1^ CNS analyzer (Carlo Erba NA 1500, Rodana).

^2^ C in CaCO_3_, determined with thermogravimetric analysis (TGA-601, Leco Corporation), was subtracted.

^3^ Used as model input values for CENTURY simulation.

^4^ Estimated based on top layer characteristics of the soil physical unit, derived from 1∶50,000 soil map.

### Soil fertility measures

Dissolved N (N–NO_3_ + N–NH_4_, mg N kg^−1^ soil) and dissolved P (P–PO_4_, mg P kg^−1^ soil) were measured to indicate the short-term availability of N and P, respectively. Two different extraction methods were used to measure dissolved P: ammonium lactate-acetic acid (ALA) extraction and the Olsen extraction. Therefore, we converted the values estimated with the ALA extraction to be comparable to those with Olsen extraction, using an empirically derived relationship [13] (Olsen-P = 2.35+0.45 ·ALA-P). The potential influence of using two different extraction methods is tested in Fig. S1 in Appendix S1. Olsen extraction is meant for neutral to alkaline soils; however, this method was also used for some of our acid (pH<5) soils. The potential interference of these acid soils on our results is tested in Fig. S2 in Appendix S2.

As a longer-term indication of N availability, we measured in-situ N mineralization rates in the mid growing season (‘summer Nmin’) for 6 weeks (datasets 1 and 3) or 8 weeks (dataset 2) (see [14] for details about the method). The difference in dissolved N between the beginning and the end of the incubation period was considered as mineralized N from organic N. Mineralized N was expressed as rates per week (mg N kg^−1^ soil week^−1^). Because denitrification in wet soil cores could have caused a considerable loss of N from the incubation tubes [14], we corrected for the N loss by adding modelled denitrification rates simulated by DAYCENT [15] to the measured N mineralization rates.

Additionally, to estimate nutrient availability for much longer terms, we used a modified version of a SOM model, CENTURY [14]. The CENTURY model simulates decomposition of soil organic C and associated flows of organic N and P. Soil total C, N, and P, soil texture, temperature, and moisture of the top soil were used as model input values. Soil temperature and moisture were simulated with a hydrological model SWAP [16]. The daily groundwater level of each site, required for the SWAP simulation, was estimated by temporal inter- and extrapolation of the observed groundwater levels in nearby wells (mostly within 30 m from the plot) using MENYANTHES software [17]. With the CENTURY model, cumulative net mineralization rates of N and P were estimated for the year of the sampling (from the first of January [mid-winter] until the end of August [end of summer], as plant traits were measured by then) (‘annual Nmin’, mg N kg^−1^ soil 243-day^−1^ and ‘annual Pmin’, mg P kg^−1^ soil 243-day^−1^) and for the preceding five years (from September five years before the sampling year to August of the sampling year) (‘5yr Nmin’, mg N kg^−1^ soil 5-year^−1^ and ‘5yr Pmin’, mg P kg^−1^ soil 5-year^−1^). Transformation processes of mineralized N and P (e.g., nitrification, denitrification, adsorption and precipitation of inorganic P) were not considered.

Finally, as very rough measures of soil fertility in the long time span, we used soil total N and P (% of total soil mass), soil N:C ratio, and soil P:C ratio.

### Plant traits

N and P concentration in leaves (LNC and LPC, respectively; mg g^−1^) were determined for dataset 3 only (Table 1). Nutrient concentrations were measured in each site for dominant species, and weighted averages (weighted by species' relative cover) were used as the plot-mean LNC and LPC. The dominant species were sampled until the cover sampled exceeded 50% of the total vascular plant cover. Note that the 50% of coverage being sampled is rather low compared to the recommended threshold of 80% [18], and therefore the LNC and LPC values in our study may slightly deviate from the true plot-mean values.

The entire above-ground plant biomass (i.e. all vascular plants of the community together) was sampled to determine whole-canopy N and P concentrations in each plot (WNC and WPC, respectively; mg g^−1^) for datasets 1 and 2 (Table 1). Since WNC and WPC reflect the aggregated characteristics of the community, they are considered as plot-mean trait values. One plot in dataset 1, which did not have nutrient concentration data, was excluded from all analyses concerning WNC and WPC. Woody species were included in WNC and WPC when they were young seedlings less than one year old, so that WNC and WPC reflect the annual uptake of the nutrients. LNC, LPC, WNC, and WPC were log-transformed prior to analyses to correct for the right-skewed distributions.

Combinations of multiple plant traits are constrained by physiological trade-offs [19]; thus, integrative traits (e.g. nutrient use efficiency) or strategy types help to express trait variability in fewer dimensions. Here we used two types of integrative plant traits: one based on species occurrences in different habitats (i.e. indicator values for nutrients, IV_nut_ [20]), and the other based on life history traits (Grime's CSR strategy [21]).

IV_nut_ is comparable to the Ellenberg indicator value for nutrients, but is tailored for Dutch flora and has a continuous scale ranging from 1 (prevailing at nutrient poor sites) to 3 (prevailing at nutrient rich sites) [20]. Plot-mean IV_nut_ values were computed as arithmetic means of IV_nut_ for each site, rather than weighted means of IV_nut_ based on species abundance, as the former was shown to be sufficient for this trait with ordinal-scale values [22]. 26 species (out of total 372 species recorded in our study), for which IV_nut_ value was not available, were excluded from the calculation of plot-mean IV_nut_ values. The trait coverage (i.e. percentage of species with IV_nut_ values within each plot) ranged from 76% to 100% (median 94%).

The CSR scheme represents the adaptive strategy of plant species along gradients of resource availability, stress, and disturbance, expressed with three axes of primary components: C (‘Competitors’), S (‘Stress tolerators’), and R (‘Ruderals’). Each species can be classified into one out of 19 classes with different combinations of strategy components, e.g. C, SR/R, or CSR. We retrieved CSR strategies from Hunt et al. [23] (313 species), supplemented by the BioFlor database [24] (35 species). For 11 species, we assigned the CSR strategy according to the method of Hodgson et al. [25], using seven life-history traits of these species retrieved from the LEDA database [26]. The remaining 13 species, for which we could not attribute CSR strategy due to lack of trait information, were excluded from the calculation of plot-mean CSR values. For each species, scores of each primary component (C, S, and R) were determined from its proportional contribution to a specific strategy (e.g. C scores of C, CS, CSR strategy are 1, 0.5, and 0.33, respectively: cf. [27]). Subsequently, for each site, plot-mean scores of C, S, and R (again, not weighted average by species abundance) were calculated. The trait coverage ranged from 86% to 100% (median 100%).

### Statistics

#### Variation partitioning

Soil fertility measures were correlated, especially strongly within the group of N-related measures and that of P-related measures (table S1). In order to examine the relative contribution of soil N and soil P to plant trait variation among sites, we partitioned the variance of each plant trait *t* (**T** *t*, a vector of *n* plots) into unique and shared effects of the two groups of predictor variables; i.e. to N-related fertility measures (**N**, a matrix of *n* x *p*_N_, where *p*_N_ is the number of N-related fertility measures, *p*_N_ = 6) and P-related fertility measures (**P**, a matrix of *n* x *p*_P_, where *p*_P_ is the number of P-related fertility measures, *p*_P_ = 5) [28]. The fraction of variance explained was indicated by the coefficient of determination of linear regression analysis for **T** *t* regressed by ***X***, where ***X*** is either **N**, **P**, or **N**&**P**. In order to correct for the different number of fertility measures within each group, we used adjusted R^2^, R^2^_(**T***t*|***X***) adj_, according to [29]. The unique effects of **N** were calculated as the fraction of variance in **T** *t* explained by **N**&**P** minus the effects of **P** on **T** *t*: R^2^_(**T***t*|**N**&**P**) adj_ − R^2^_(**T***t*|**P**) adj_. Identically, the unique effects of **P** on **T** *t* were calculated as R^2^_(**T***t*|**N**&**P**) adj_ − R^2^_(**T***t*|**N**) adj_. Shared effects of **N** and **P** on **T** *t* were calculated as R^2^_(**T***t*|**N**) adj_ + R^2^_(**T***t*|**P**) adj_ − R^2^_(**T***t*|**N**&**P**) adj_. The analysis was performed in R [30].

Additionally, we tested if N-related fertility measures explain significantly more variance in plot-mean traits than P-related fertility measures, and vice versa, following the bootstrapping method described in [29] using R [30]. Bootstrapped adjusted R^2^ was computed 1000 times, and the difference between the adjusted R^2^ between groups, D*_i_*  =  R^2^_(**T***t*|**N**) adj-boot,*i*_ − R^2^_(**T***t*|**P**) adj-boot,*i*_, was calculated for each *i*th bootstrapping. *p*-values were calculated from the distribution of D*_i_*, as the fraction of D*_i_* that falls below zero (when the median of D*_i_* was positive, i.e. variance explained by **N** was larger than that by **P**) or above zero (when the median of D*_i_* was negative).

#### Hierarchical partitioning

In order to examine the most relevant timescale of soil fertility for explaining the variation of plant traits, we used the hierarchical partitioning method [31], [32]. This method allows, within the hypothetical relationship between trait variance (response variable) and *k* fertility measures with different timescales (predictor variables), to quantify the independent contribution of a fertility measure *S* to the explained variance of a trait without being confounded by the other *k*-1 fertility measures. The hierarchical partitioning method computes the increase in goodness-of-fit when *S* is added to the model (in our case: a linear multivariate regression model of a trait regressed by fertility measures) compared to the model without *S*, and averages the increase over all possible models that include *S* as a predictor variable (i.e. 2*^k^* models). In this way, the variation of a trait explained by *S* is divided into an independent effect of *S* and joint effect of *S* with other fertility measures. Negative values of a joint effect mean that the interactive effects of *S* and the other fertility measures on the trait are suppressive, rather than enhancing. An advantage of using the hierarchical partitioning over a one-model approach is that the averaging eliminates the problem of multicollinearity among predictor variables [32]. We conducted hierarchical partitioning separately for N-related and P-related fertility measures (i.e. *k* = *p*_N_ or *k* = *p*_P_), so that interactions of N and P do not obscure the effects of timescale. We used R^2^ as the goodness-of-fit measure of the models. The analysis was performed in R [30] with the package ‘hier.part’ [33].

Foe each fertility measures, the statistical significance of their independent contribution to a plant trait was tested by randomizing the pairs of trait and fertility values for 1000 times [32] in R [30]. Z-scores were calculated from the generated distribution of randomized independent contributions, and statistical significance was determined based on the upper 95% confidence limit (Z≥1.65).

In addition, independent contributions were compared among fertility measures with different timescales by means of bootstrapping in R [30]. 134 plots were randomly selected with replacement, and the 95% confidence interval was computed from the distribution of independent contributions of 1000x bootstrapped 134 plots. Furthermore, the difference in independent effects between two fertility measures was computed for all combinations. When more than 95% of the difference was larger or smaller than zero, we considered that the two fertility measures had significantly (*p*<0.05) different magnitudes of independent effects.

#### Principal component regression

Since the soil fertility measures were strongly correlated, we extracted the main axes of variation by a Principal Component Analysis (PCA) in R [30], based on a correlation matrix to correct for differences in metrics among variables. We used six soil N measures, five soil P measures, and soil N:P ratio. All these variables were log-transformed prior to the analysis. We extracted the scores of sites along PCA axis 1 and 2 and related them to plot-mean values of each trait using linear regression.

## Results

### Relative contributions of soil N and P measures to plot-mean plant traits

**Figure 1:**
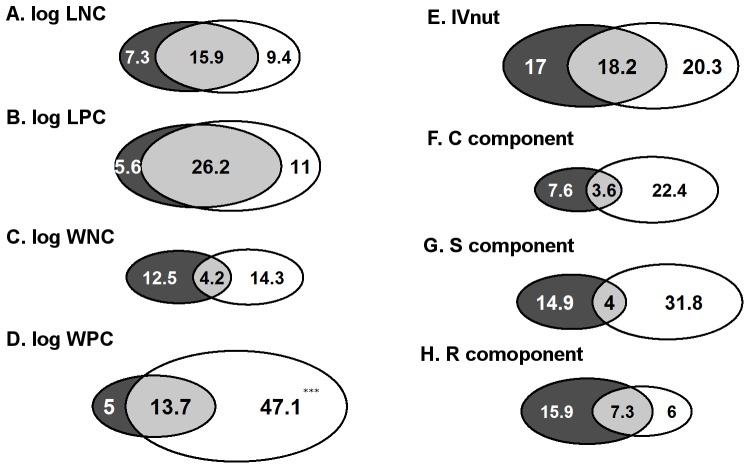
Partitioning of among-site plant trait variation. Trait variations (in percentage of variance) are divided into unique effects of soil N measures (dark grey), unique effects of soil P measures (white), and shared effects of both (light grey). Examined plot-mean plant traits are A: log-transformed leaf N concentration, LNC (mg/g) (*n* = 51), B: log-transformed leaf P concentration, LPC (mg/g) (*n = *51), C: log-transformed N concentration of above-ground plant biomass, WNC (mg/g) (*n = *82), D: log-transformed P concentration of above-ground plant biomass, WPC (mg/g) (*n = *82), E: indicator value for nutrients, IV_nut_ (*n = *134), F: C component (*n = *134), G: S component (*n = *134), and H: R component (*n = *134). When the contribution of N or P measures to the total explained variance is significantly larger than the other, this is indicated by asterisks (^***^: *p*<0.001).

Bivariate correlations between plant traits and soil fertility measures were often significant (table S2). All soil fertility measures together explained less than half of the among-site variation in leaf nutrient concentration (32.7% for LNC [Fig. 1A] and 42.8% for LPC [Fig. 1B]), in which a large part was attributed to the shared effects of soil N and P measures. There was no significant difference between soil N and P measures in their contribution to the total explained trait variance (*p* = 0.42 for LNC, *p* = 0.30 for LPC). P concentrations at the whole-canopy level were more strongly related to soil fertility measures than those on a leaf level (i.e. 65.9% of variance in WPC was explained, Fig. 1D). In contrast, the relationships between N concentrations and soil fertility were weak both on a whole-canopy level and on a leaf level (i.e. 31.0% of variance in WNC was explained, Fig. 1C). For both WPC and WNC, the shared effects of soil N and P measures were relatively small, and the contribution of soil P measures was larger than that of soil N measures (significant for WPC [*p*<0.001] but not for WNC [*p* = 0.42]).

55.5% of the variance in IV_nut_ was explained by soil fertility measures (Fig. 1E), of which less than half was attributed to the shared effects of soil N and P measures (18.2%). The contribution of soil P and N measures was not significantly different (*p* = 0.33).

Of the three components of the CSR strategy, soil fertility measures explained the variance in the S component to a largest extent (50.7%, Fig. 1G), followed by the C component (33.6%, Fig. 1F) and the R component (29.3%, Fig. 1H). Shared effects contributed to a small proportion of the explained variance (4.0% for S, 3.6% for C, and 7.3% for R). For the R component, the contribution of soil N measures was almost significantly (*p* = 0.057) larger than that of soil P measures, whereas for the C and S components, the contribution of soil P measures was considerably (but not significantly, *p* = 0.16 and *p* = 0.21, respectively) larger than that of soil N measures.

### Effects of different timescales of soil fertility measures on plot-mean plant traits

**Figure 2:**
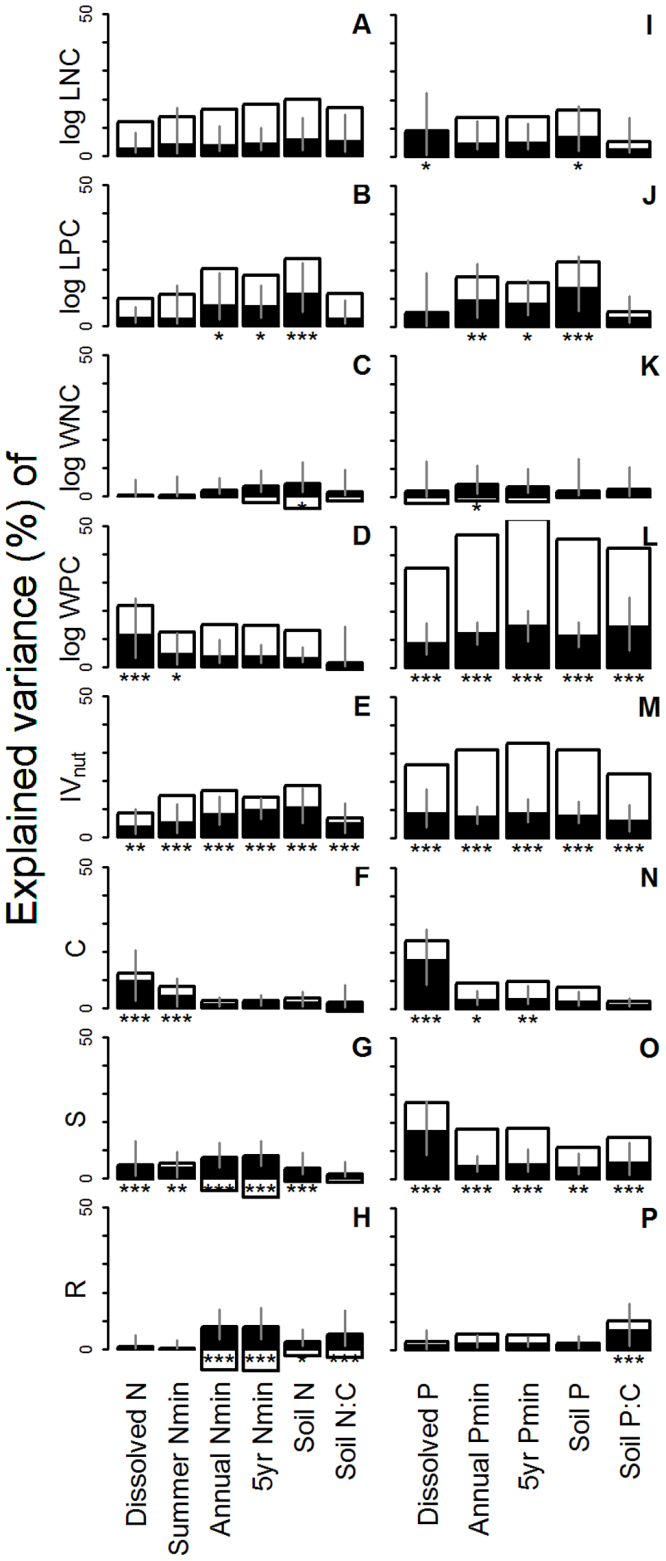
Hierarchical partitioning of among-site plant trait variation. Trait variations are divided into independent effects of a soil fertility measure (black bars) and its joint effects with other measures (white bars). All fertility measures (see Table 1 for specification) were log-transformed prior to the analysis. Examined plot-mean plant traits are A&I: log LNC, B&J: log LPC, C&K: log WNC, D&L: log WPC, E&M: IV_nut_, F&N: C component, G&O: S component, and H&P: R component. See the caption of Figure 1 for the specification and sample number of each plant trait. Computation was done within each group of fertility measures (i.e. within soil N measures [A–H] and soil P measures [I–P]). Asterisks indicate that the independent effect was significant, based on Z-scores computed with randomization (^*^: *p*<0.05, ^**^: *p*<0.01, ^***^: *p*<0.001). 95% confidence intervals of independent effects, obtained by 1000-time bootstrapping, are shown.

Only few fertility measures had significant independent effects on LNC and WNC (i.e. dissolved P and soil total P for LNC [Fig. 2I], soil total N and annual Pmin for WNC [Fig. 2C and 2K]). The independent effects were not significantly (*p*>0.05) different among fertility measures with different timescales (Table S3).

For LPC, independent effects were significant for three out of six soil N measures (Fig. 2B) and for three out of five soil P measures (Fig. 2J). Longer-term measures (e.g. soil total N, soil total P) tended to have larger independent effects, but the differences with short-term measures were not very apparent (e.g. only dissolved N had slightly smaller effects than longer-term measures i.e. annual Nmin, 5yr Nmin, and soil total N, *p*<0.05, Table S3).

For WPC, all soil P measures had significant independent effects (Fig. 2L). Longer-term measures (e.g. 5yr Pmin and soil P:C ratio) tended to have larger independent effects, but the differences were not significant (*p*>0.05) except between 5yr Pmin and annual Pmin (*p*<0.01, Table S3).

For IV_nut_, independent effects were significant for all fertility measures (Fig. 2E, Fig. 2M). The independent effects were not significantly (*p*>0.05) different among fertility measures with different timescales except between 5yr Pmin and annual Pmin (*p*<0.05, Table S3).

For the C component of the CSR strategy, independent effects were significant and stronger for shorter-term fertility measures (e.g. dissolved N, summer Nmin, dissolved P; Fig. 2F and Fig. 2N). In contrast, for the S component, almost all fertility measures had significant independent effects (Fig. 2G, Fig. 2O), but the difference was less apparent (although dissolved P had stronger independent effects than all other soil P measures, *p*<0.05, Table S3). The strong independent effects of dissolved P on the C and S components could be an artefact of using Olsen extraction methods for some of the acid soils (see Appendix S2 for details). For the R component, soil P measures hardly had significant independent effects, and timescale did not matter (*p*>0.05, Table S3) (Fig. 2P), whereas intermediate-term soil N measures (e.g. annual Nmin, 5yr Nmin) had significant and stronger (*p*<0.01) independent effects than shorter-term measures (e.g. dissolved N, summer Nmin) (Fig. 2H, Table S3).

### Relations between integrated soil fertility measures and plot-mean plant traits

**Figure 3:**
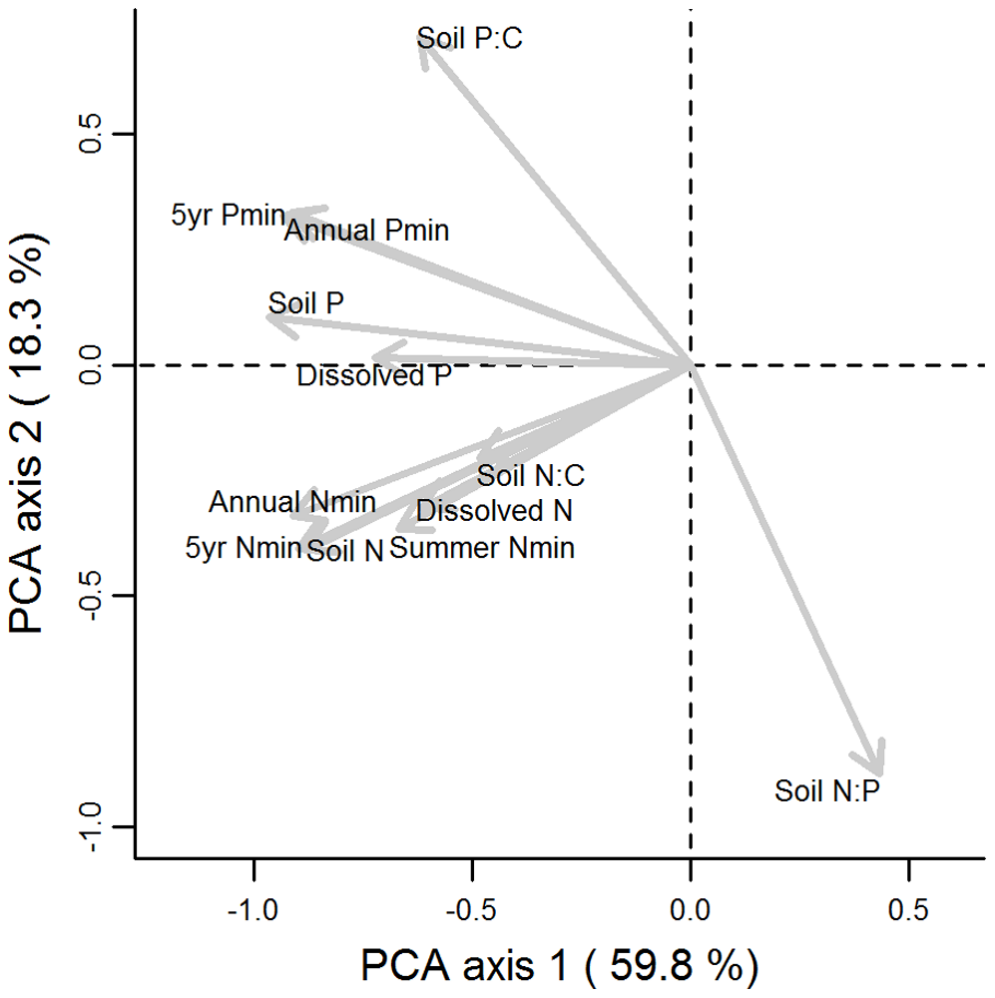
Principal Component Analysis (PCA) of 12 soil fertility measures for 134 plots.

PCA analysis extracted major axes of variation in soil fertility measures. The first axis, explaining 59.8% of the variance, was related to overall nutrient availability of a site (i.e. high in both soil N and P), with the negative axis values representing fertile conditions (Fig. 3). The second axis, explaining 18.3% of the variance, separated relatively P-rich (positive axis values) from N-rich (negative axis values) sites (Fig. 3).

**Figure 4:**
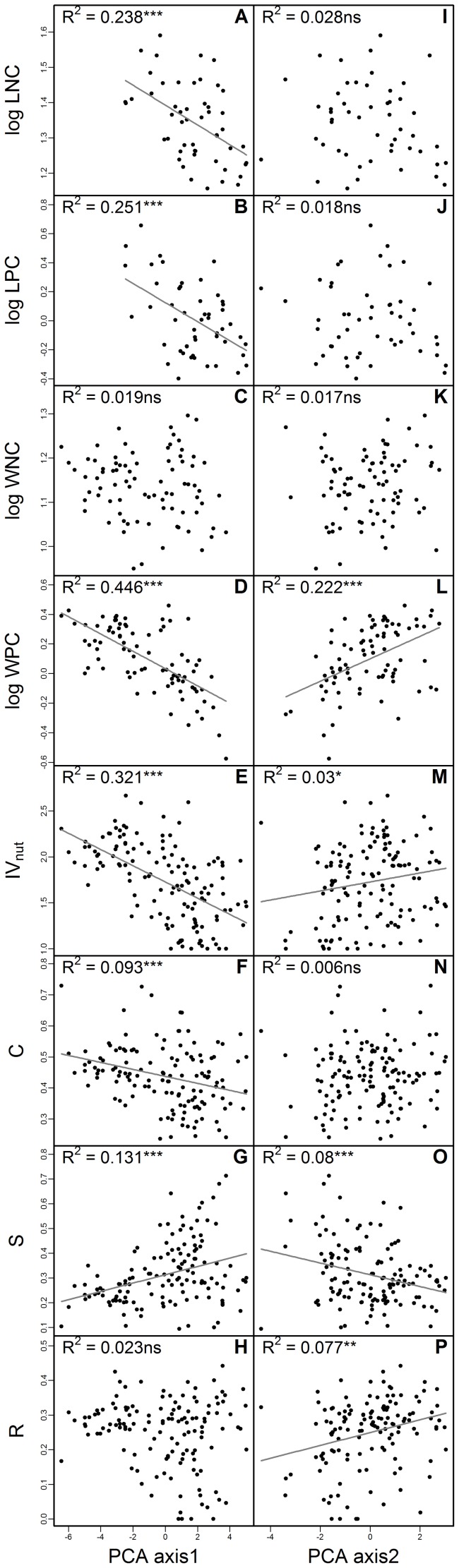
Relationships of the first and second PCA axis with plot-mean plant traits. R^2^ and *p*-values of linear regression analysis are shown. Lines represent the regression model (only when *p*<0.05). See the caption of Figure 1 for the specification and sample numbers for each plant traits.

Most plant traits, except WNC and the R component, were significantly related to PCA axis 1 (Fig. 4A–H). PCA axis 2 was significantly related only to WPC, IV_nut_, the S component, and the R component (Fig. 4I–P). Multiple regression analysis showed that both PCA axes had significant (*p*<0.05) effects for WPC, IV_nut_, and the S component (and almost significant [*p*<0.07] effects for the R component); this indicates that the two axes had complementary rather than redundant effects on these traits.

## Discussion

### Mutual and stoichiometric control of soil N and P on plant traits

In line with previous studies [2], [4], [5], we found that fertile soils are associated with plant communities composed of species with higher nutrient concentrations, and include more competitor rather than stress-tolerant types of species. However, we highlight the importance of considering both soil N and soil P concentrations in explaining trait variation among communities. Indeed, we found that soil N and P had a strong mutual (shared) effect on LNC and LPC while other traits tend to be related to unique effect of soil N or soil P.

There are several possible mechanisms that explain why LNC is related to both soil N and soil P (rather than only to soil N) and why LPC is related to both soil N and soil P (rather than only to soil P). Although leaf-level nutrient concentrations change plastically with changing concentrations of that element in the soil [34], LNC and LPC are strongly coupled with a 2/3-power law of scaling [35], [36] due to the mutually dependent roles of N and P in photosynthesis. Consequently, rapid growth, which is associated with high LNC and high LPC [35], is realized only when both soil N and P availability for plants is high. This clarifies the observed pattern that the shared effects of soil N and P (i.e. overall fertility effects), rather than the unique effects of soil N and soil P, explain the large variation in LNC and LPC.

In contrast, the nutrient concentrations on a whole-canopy level (WNC and WPC) reflect the availability of single elements more strongly than on a leaf level, because plants store excess elements in non-leaf organs. This is reflected in a much larger variation in N:P ratio in non-leaf organs than in leaves [37]. The predictive power of soil P on WPC was especially strong, because plants can adjust P concentrations much more flexibly than N concentrations to the nutrient availability in the soil [38].

For IV_nut_, the unique effects of single elements contributed to most of the explained variance, although the shared effect of soil N and P also accounted for a large part of the variance. For the CSR strategy, most of the explained variance was related to the unique effects of single elements, rather than to the shared effects of soil N and P. Here, the effects of N and P were highly different, indicating a stoichiometric control of soil N and P on the CSR strategy of vegetation. The unique effects of soil P were larger than those of soil N for the C and S components, in accordance with the findings of Ceulemans et al. [39] based on Olsen-extracted P availability and KCl-extracted N availability. Variance in the R component was least explained, reflecting that environmental drivers other than nutrient availability (i.e. disturbance) are the primary determinants of the distribution of ruderals [21].

To test whether the above-mentioned trends were merely an artefact of biased distribution of N- and P-limited ecosystems in our study, we conducted the same analyses for N-limited and P-limited plots separately (Appendix S3). When only N-limited plots were considered, contributions of soil N measures to plant trait variance tended to increase slightly (Fig. S3 left); however, other major trends (e.g. a stronger effect of soil P on WPC than that of soil N [*p* = 0.094], small shared effects on CSR strategy) remained. When only P-limited plots were considered, the contributions of soil P remained mostly unchanged or even decreased for some traits (Fig. S3 right). It is particularly notable that the stronger determinants for stress tolerators were N-related fertility measures in N-limited plots (*p*<0.05) and P-related fertility measures in P-limited plots (*p* = 0.079) (Fig. S3). Furthermore, signs of correlations between soil N measures and integrative plant traits (IV_nut_, CSR strategy) reversed in P-limited plots compared to N-limited plots for most cases (e.g. stress tolerator increased as soil N availability increased in P-limited plots only; see Table S4 in comparison to Table S5 in Appendix S3). These reversed relationships imply that, under P-poor conditions, high soil N availability results in an extreme imbalance of soil N and P and therefore induces harsher environments for plant species. This emphasizes the importance of explicitly considering stoichiometric effects of nutrients on plant functioning.

### Timescale has only minor impact on fertility–trait relationships

Contrary to previous results [2], [10], we found no clear indication that the fertility–trait relationship is sensitive to the timescale of the soil fertility measure. Almost all soil fertility measures were closely correlated, and therefore the independent effects of a particular fertility measure were almost never outstandingly stronger than that of others.

It is more difficult to estimate the availability of soil P for plants than that of soil N; this is because various geochemical processes of soil inorganic P, such as adsorption and precipitation, are involved. These processes were not explicitly included in our soil P measures, possibly obscuring the impact of timescale on the fertility–trait relationships. To test this, we examined for a subset of our dataset (36 plots) whether adding extra measurements of soil P availability improved the fertility–trait relationships (Appendix S4). Neither summer gross P mineralization rates (i.e. increase in Olsen-extractable P [both inorganic and organic] in in-situ incubation experiments), oxalate-extractable P (i.e. an estimate of reducible amount of P, which includes P adsorbed on Al- and Fe-hydroxides and oxides [40]), nor the degree of phosphate saturation (i.e. the percentage of oxalate-extracted P over half of oxalate-extracted Al plus Fe, an index for soil capacity to release P [41]) were superior to other P fertility measures in explaining trait variations (Fig. S5 in Appendix S4). So, the virtual absence of timescale impacts on soil fertility–trait relationships was not likely due to the inadequacy of selected P measures.

### Use of integrated soil fertility measures to explain community trait composition

There was no single fertility measure that dominantly explained plant trait variations; this indicates that plant traits are mutually controlled by multiple soil fertility measures, suggesting the usefulness of using integrated fertility measures. Indeed, the main axis of variation in fertility measures, overall fertility gradient (PCA axis 1), was related to almost all plant traits. However, the explanatory power of the PCA axis 1 (i.e. R^2^ values in Fig. 4) was only slightly higher for LNC and LPC than that of the best single fertility measures (i.e. variance explained by a fertility measure, including both independent and joint effects, in Fig. 2). For the other traits, PCA axis 1 explained less than the best single fertility measures. The other integrated fertility measure, the relative availability of soil N and P (PCA axis 2), had small but complementary effects for some plant traits (i.e. WPC, IV_nut_, S component, R component). Similarly, including the type of nutrient limitation of plants (i.e. N- or P-limited) improved the relationship between overall fertility and several plant traits (WNC, WPC, IV_nut_, R component) (Fig. S4 in Appendix S3). This means that the fertility–trait relationships are modulated by the type and magnitude of nutrient limitation, probably because the most influential factor of fertility is not identical for all sites but depends on which nutrient is actually limiting the plant growth of the site.

These findings imply that simultaneous consideration of overall fertility and N:P stoichiometry (either in soil or in plants) is a prerequisite for improving fertility–trait relationships. Note that the N:P stoichiometry effect cannot be assessed by individual fertility measures alone, but it can be explicitly tested by integrated fertility measures (i.e. PCA axis 2 in our case). This suggests the appropriateness of using integrated fertility measures as a starting point to explore which aspect of soil fertility has a relevant effect on the specific plant trait.

### Wrong choice of fertility measure, or intrinsically moderate relationships between fertility and plant traits?

As in previous studies [2], [4], variance of LNC was less strongly explained by fertility measures than that of LPC in our study, even if various types of fertility measures were considered (i.e. 32.7% explained by all fertility measures together, 19.9% by the best single measure [soil total N] and 23.8% by the best integrated measure [PCA axis 1]). Also, only a minor part of the variation was explained by soil fertility for WNC (31.0%, 3.2%, and 1.9%, respectively) and for R component (29.3%, 6.7%, and 7.7%, respectively). In contrast, several other plant traits could be well explained by fertility measures: 65.9%, 52.6%, and 44.6% for WPC, respectively, and 55.5%, 33.6%, and 32.1% for IV_nut_, respectively. Reasonably good relationships between IV_nut_ or equivalent (e.g. Ellenberg indicator value for nutrients) and a single soil fertility measure have also been observed before (e.g. 49% explained by annual N mineralization rates [5], 35% explained by ‘nitrification degree’ [9], 29% explained by oxalate-extractable P [42]).

Thus, the moderate fertility–trait relationships of some plant traits are most likely not because of a wrong choice of fertility measure but because of the intrinsic nature of the relationships for these traits as explained earlier. Furthermore, environmental factors other than soil fertility also influence plant traits. For example, the occurrence of ruderal species (R component of CSR strategy) is primarily associated with disturbance rather than soil fertility [21], and LNC and LPC are weakly but consistently related with drought and oxygen stress [43]. Since plant traits are coordinated through physiological trade-offs and the coordination of the traits is strongly modulated by environmental factors [19], [44], simultaneous consideration of multiple environmental factors is necessary to improve the prediction of these traits. Reasonably good relationships between fertility and IV_nut_ encourage the application of this relationship in species-distribution models to predict the functional composition of plant species. Also, other plant traits unexamined in our study, such as specific leaf area, may be considered for examining fertility–trait relationships. Specific leaf area was, however, not better related to soil fertility measures than LPC [4].

In conclusion, our study showed that among-site variations in nutrient-related plant traits are consistently, although moderately for some traits, related to soil fertility measures. Whether a trait has only a moderate relationship depends on the mechanism through which soil fertility and other factors regulate the trait variation. The timescale of the fertility measure has only negligible or minor effects on fertility–trait relationships, whereas the mutual and/or stoichiometric effects of N and P should be considered to improve the relationships. Since the relative importance of soil fertility measures is different among plant traits, a scan of integrated fertility measures will facilitate identification of influential fertility measures (or groups of fertility measures) for each specific plant trait separately.

## Supporting Information

Table S1 Correlations among soil fertility measures.
(DOCX)

Table S2 Correlations between plot-mean plant traits and soil fertility measures.
(DOCX)

Table S3 Comparison of 1000x bootstrapped independent effects among soil fertility measures.
(DOCX)

Appendix S1 **Testing the effects of using two extraction methods for dissolved P.**
(DOCX)

Appendix S2 **Testing the effects of using Olsen-P extraction for acid soils.**
(DOCX)

Appendix S3 **Fertility–trait relationships in N- and P-limited ecosystems.**
(DOCX)

Appendix S4 **Testing additional soil P measures for a subset of dataset.**
(DOCX)
