# Supplementary material for: Relationships between Nutrient-Related Plant Traits and Combinations of Soil N and P Fertility Measures
Source: PLoS One. 2013 Dec 31;8(12):e83735. doi: 10.1371/journal.pone.0083735 (PMC3877083; doi:10.1371/journal.pone.0083735)
Supplement: Table S1 — Correlations among soil fertility measures. (DOCX) [file pone.0083735.s001.docx]

**Table S1.** Correlations among soil fertility measures (*n*=134). All fertility measures were log-transformed prior to the analysis. Pearson’s correlation coefficient and *p*-values are shown (*: *p* <0.05, **: *p* <0.01%, ***: *p* <0.001, ns: not significant). Correlation coefficients larger than 0.7 are shown with bold letters.

|  | Dissolved N | Summer N | Annual Nmin | 5yr Nmin | Soil N | Soil N:C | Dissolved P | Annual Pmin | 5yr Pmin | Soil P | Soil P:C |
| --- | --- | --- | --- | --- | --- | --- | --- | --- | --- | --- | --- |
| Dissolved N |  |  |  |  |  |  |  |  |  |  |  |
| Summer Nmin | 0.45 *** |  |  |  |  |  |  |  |  |  |  |
| Annual Nmin | 0.57 *** | 0.66 *** |  |  |  |  |  |  |  |  |  |
| 5yr Nmin | 0.61 *** | 0.67 *** | **0.98** *** |  |  |  |  |  |  |  |  |
| Soil N | 0.58 *** | 0.68 *** | **0.94** *** | **0.97** *** |  |  |  |  |  |  |  |
| Soil N:C | 0.20 * | 0.24 ** | 0.53 *** | 0.58 *** | 0.49 *** |  |  |  |  |  |  |
| Dissolved P | 0.55 *** | 0.51 *** | 0.59 *** | 0.56 *** | 0.57 *** | 0.06 ns |  |  |  |  |  |
| Annual Pmin | 0.44 *** | 0.47 *** | **0.74** *** | 0.69 *** | 0.66 *** | 0.31 *** | 0.68 *** |  |  |  |  |
| 5yr Pmin | 0.46 *** | 0.48 *** | **0.75** *** | **0.71** *** | 0.69 *** | 0.36 *** | 0.67 *** | **0.98** *** |  |  |  |
| Soil P | 0.51 *** | 0.60 *** | **0.85** *** | **0.84** *** | **0.87** *** | 0.39 *** | 0.65 *** | **0.90** *** | **0.93** *** |  |  |
| Soil P:C | 0.13 ns | 0.15 ns | 0.33 *** | 0.29 *** | 0.25 ** | 0.42 *** | 0.33 *** | **0.75** *** | **0.78** *** | 0.66 *** |  |
| Soil N:P | -0.04 ns | -0.04 ns | -0.09 ns | -0.02 ns | -0.02 ns | 0.06 ns | -0.33 *** | -0.66 *** | -0.67 *** | -0.52 *** | **-0.88** *** |
