# Supplementary material for: Relationships between Nutrient-Related Plant Traits and Combinations of Soil N and P Fertility Measures
Source: PLoS One. 2013 Dec 31;8(12):e83735. doi: 10.1371/journal.pone.0083735 (PMC3877083; doi:10.1371/journal.pone.0083735)
Supplement: Table S2 — Correlations between plot-mean plant traits and soil fertility measures. (DOCX) [file pone.0083735.s002.docx]

**Table S2.** Correlations between plot-mean plant traits and soil fertility measures. All fertility measures and LNC, LPC, WNC, and WPC were log-transformed prior to analysis. Pearson’s correlation coefficient and *p*-values are shown (*: *p* <0.05, **: *p* <0.01%, ***: *p* <0.001, ns: not significant).

|  | LNC | LPC | WNC | WPC | IV_nut_ | C | S | R |
| --- | --- | --- | --- | --- | --- | --- | --- | --- |
| Dissolved N | 0.35 * | 0.31 * | 0.07 ns | 0.47 *** | 0.29 *** | 0.35 *** | -0.21 * | -0.08 ns |
| Summer Nmin | 0.37 ** | 0.34 * | 0.02 ns | 0.35 ** | 0.38 *** | 0.28 ** | -0.23 ** | 0.02 ns |
| Annual Nmin | 0.41 ** | 0.45 *** | 0.14 ns | 0.39 *** | 0.41 *** | 0.16 ns | -0.17 * | 0.06 ns |
| 5yr Nmin | 0.42 ** | 0.42 ** | 0.13 ns | 0.38 *** | 0.38 *** | 0.16 ns | -0.12 ns | -0.01 ns |
| Soil N | 0.45 ** | 0.49 *** | 0.06 ns | 0.36 *** | 0.43 *** | 0.19 * | -0.16 ns | 0.01 ns |
| Soil N:C | 0.41 ** | 0.34 * | 0.00 ns | 0.06 ns | 0.26 ** | -0.10 ns | -0.03 ns | 0.14 ns |
| Dissolved P | 0.30 * | 0.22 ns | 0.01 ns | 0.59 *** | 0.51 *** | 0.49 *** | -0.52 *** | 0.17 ns |
| Annual Pmin | 0.37 ** | 0.42 ** | 0.18 ns | 0.68 *** | 0.56 *** | 0.30 *** | -0.42 *** | 0.23 ** |
| 5yr Pmin | 0.37 ** | 0.40 ** | 0.14 ns | 0.73 *** | 0.58 *** | 0.31 *** | -0.42 *** | 0.23 ** |
| Soil P | 0.41 ** | 0.48 *** | 0.15 ns | 0.67 *** | 0.56 *** | 0.28 ** | -0.33 *** | 0.15 ns |
| Soil P:C | 0.22 ns | 0.23 ns | 0.17 ns | 0.65 *** | 0.48 *** | 0.16 ns | -0.38 *** | 0.32 *** |
| Soil N:P | 0.03 ns | -0.02 ns | -0.19 ns | -0.70 *** | -0.39 *** | -0.23 ** | 0.40 *** | -0.28 ** |
|  |  |  |  |  |  |  |  |  |
| N. of plots | 51 | 51 | 82 | 82 | 134 | 134 | 134 | 134 |
