# Supplementary material for: Relationships between Nutrient-Related Plant Traits and Combinations of Soil N and P Fertility Measures
Source: PLoS One. 2013 Dec 31;8(12):e83735. doi: 10.1371/journal.pone.0083735 (PMC3877083; doi:10.1371/journal.pone.0083735)
Supplement: Table S3 — Comparison of 1000x bootstrapped independent effects among soil fertility measures. (DOCX) [file pone.0083735.s003.docx]

**Table S3.** Comparison of 1000x bootstrapped independent effects among soil fertility measures. “+” means that the independent effect of the fertility measure was larger than that of the counterpart for 95% of the bootstrapped cases. “-” means it was smaller. *p*-values of the difference are given (*: *p* <0.05, **: *p* <0.01%, ***: *p* <0.001, ns: not significant).

|  |  | Compared with | | | | | |  |  | Compared with | | | | |
| --- | --- | --- | --- | --- | --- | --- | --- | --- | --- | --- | --- | --- | --- | --- |
| Trait | Soil N measure | Dissolved N | Summer Nmin | Annual Nmin | 5yr Nmin | Soil N | Soil N:C |  | Soil P measure | Dissolved P | Annual Pmin | 5yr Pmin | Soil P | Soil P:C |
| LNC | Dissolved N |  |  |  |  |  |  |  | Dissolved P |  |  |  |  |  |
|  | Summer Nmin | ns |  |  |  |  |  |  | Annual Pmin | ns |  |  |  |  |
|  | Annual Nmin | ns | ns |  |  |  |  |  | 5yr Pmin | ns | ns |  |  |  |
|  | 5yr Nmin | ns | ns | ns |  |  |  |  | Soil P | ns | ns | ns |  |  |
|  | Soil N | ns | ns | ns | ns |  |  |  | Soil P:C | ns | ns | ns | ns |  |
|  | Soil N:C | ns | ns | ns | ns | ns |  |  |  |  |  |  |  |  |
| LPC | Dissolved N |  |  |  |  |  |  |  | Dissolved P |  |  |  |  |  |
|  | Summer Nmin | ns |  |  |  |  |  |  | Annual Pmin | ns |  |  |  |  |
|  | Annual Nmin | +* | ns |  |  |  |  |  | 5yr Pmin | ns | ns |  |  |  |
|  | 5yr Nmin | +* | ns | ns |  |  |  |  | Soil P | ns | ns | ns |  |  |
|  | Soil N | +* | ns | ns | ns |  |  |  | Soil P:C | ns | ns | ns | -* |  |
|  | Soil N:C | ns | ns | ns | ns | -* |  |  |  |  |  |  |  |  |
| WNC | Dissolved N |  |  |  |  |  |  |  | Dissolved P |  |  |  |  |  |
|  | Summer Nmin | ns |  |  |  |  |  |  | Annual Pmin | ns |  |  |  |  |
|  | Annual Nmin | ns | ns |  |  |  |  |  | 5yr Pmin | ns | ns |  |  |  |
|  | 5yr Nmin | ns | ns | ns |  |  |  |  | Soil P | ns | ns | ns |  |  |
|  | Soil N | ns | ns | ns | ns |  |  |  | Soil P:C | ns | ns | ns | ns |  |
|  | Soil N:C | ns | ns | ns | ns | ns |  |  |  |  |  |  |  |  |
| WPC | Dissolved N |  |  |  |  |  |  |  | Dissolved P |  |  |  |  |  |
|  | Summer Nmin | ns |  |  |  |  |  |  | Annual Pmin | ns |  |  |  |  |
|  | Annual Nmin | ns | ns |  |  |  |  |  | 5yr Pmin | ns | +** |  |  |  |
|  | 5yr Nmin | ns | ns | ns |  |  |  |  | Soil P | ns | ns | ns |  |  |
|  | Soil N | -* | ns | ns | ns |  |  |  | Soil P:C | ns | ns | ns | ns |  |
|  | Soil N:C | ns | ns | ns | ns | ns |  |  |  |  |  |  |  |  |
| IV_nut_ | Dissolved N |  |  |  |  |  |  |  | Dissolved P |  |  |  |  |  |
|  | Summer Nmin | ns |  |  |  |  |  |  | Annual Pmin | ns |  |  |  |  |
|  | Annual Nmin | ns | ns |  |  |  |  |  | 5yr Pmin | ns | +* |  |  |  |
|  | 5yr Nmin | ns | ns | ns |  |  |  |  | Soil P | ns | ns | ns |  |  |
|  | Soil N | ns | ns | ns | ns |  |  |  | Soil P:C | ns | ns | ns | ns |  |
|  | Soil N:C | ns | ns | ns | ns | ns |  |  |  |  |  |  |  |  |
| C | Dissolved N |  |  |  |  |  |  |  | Dissolved P |  |  |  |  |  |
|  | Summer Nmin | ns |  |  |  |  |  |  | Annual Pmin | -** |  |  |  |  |
|  | Annual Nmin | -* | ns |  |  |  |  |  | 5yr Pmin | -** | ns |  |  |  |
|  | 5yr Nmin | -* | ns | ns |  |  |  |  | Soil P | -** | ns | ns |  |  |
|  | Soil N | -* | ns | ns | ns |  |  |  | Soil P:C | -*** | ns | -* | ns |  |
|  | Soil N:C | ns | ns | ns | ns | ns |  |  |  |  |  |  |  |  |
| S | Dissolved N |  |  |  |  |  |  |  | Dissolved P |  |  |  |  |  |
|  | Summer Nmin | ns |  |  |  |  |  |  | Annual Pmin | -** |  |  |  |  |
|  | Annual Nmin | ns | ns |  |  |  |  |  | 5yr Pmin | -* | ns |  |  |  |
|  | 5yr Nmin | ns | ns | ns |  |  |  |  | Soil P | -* | ns | ns |  |  |
|  | Soil N | ns | ns | ns | -* |  |  |  | Soil P:C | -* | ns | ns | ns |  |
|  | Soil N:C | ns | ns | -** | -** | ns |  |  |  |  |  |  |  |  |
| R | Dissolved N |  |  |  |  |  |  |  | Dissolved P |  |  |  |  |  |
|  | Summer Nmin | ns |  |  |  |  |  |  | Annual Pmin | ns |  |  |  |  |
|  | Annual Nmin | +** | +** |  |  |  |  |  | 5yr Pmin | ns | ns |  |  |  |
|  | 5yr Nmin | +** | +** | ns |  |  |  |  | Soil P | ns | ns | ns |  |  |
|  | Soil N | ns | +* | -* | -** |  |  |  | Soil P:C | ns | ns | ns | ns |  |
|  | Soil N:C | +* | +* | ns | ns | ns |  |  |  |  |  |  |  |  |
