# Supplementary material for: Relationships between Nutrient-Related Plant Traits and Combinations of Soil N and P Fertility Measures
Source: PLoS One. 2013 Dec 31;8(12):e83735. doi: 10.1371/journal.pone.0083735 (PMC3877083; doi:10.1371/journal.pone.0083735)
Supplement: Appendix S1 — Testing the effects of using two extraction methods for dissolved P. (DOCX) [file pone.0083735.s004.docx]

**Appendix S1. Testing the effects of using two extraction methods for dissolved P**

Two different extraction methods were used to estimate dissolved P (i.e. ALA extraction for dataset 2 and Olsen extraction for dataset 1 and 3), and ALA-extracted dissolved P was converted to be equivalent to Olsen-extracted dissolved P (see Method section of the main text). Since the conversion equation was merely derived from an empirical relationship of Portuguese acid soils [[1](#_ENREF_1)], possible errors during the conversion could have influenced our findings on dissolved P vs. plant traits relationships. To test the effect of extraction methods, we regressed each of the plant traits against dissolved P and extraction method (as a dummy variable of 0 and 1) in a multiple linear regression analysis. Note that also for WNC and WPC different measurement methods had been applied among datasets (i.e. CNS analyzer and Kjeldahl digestion for WNC, HNO_3_+HCl digestion and Kjeldahl digestion for WPC). However, both methods measure the total fraction of N or P with highly destructive reagents and therefore we do not expect large errors associated with these methods.

Only for the C component and the S component, the extraction method significantly (*p* <0.05) mediated the relation between dissolved P and the plant trait (Fig. S1): given a certain level of dissolved P, the C component was lower (*p* <0.001) and the S component was higher (*p* <0.05) when ALA extraction was used instead of Olsen extraction. This could be a relation confounded by other factors which were different among datasets such as plant biomass and soil pH. Sites which used ALA extraction happened to have on average higher above-ground biomass (*p* <0.001 with t-test) and lower pH (*p*=0.06 with U-test) than those which used Olsen extraction. At the same time, the C and S components were strongly correlated with biomass (positively for C and negatively for S, *p* <0.001 with Pearson’s correlation), and the S component was negatively correlated with pH (*p*<0.001 with Spearman’s correlation). We confirmed the impacts of biomass and pH with an additional multiple regression analysis of traits versus dissolved P, the extraction method for dissolved P (dummy variable), plant biomass, and soil pH for a subset of data (*n*=81) for which plant biomass data was available. The effect of the extraction method was no longer significant (*p*<0.05) for both C and S components. This implies that the choice of the extraction methods for dissolved P per se does not have a direct consequence on our findings.

**Figure S1.** Relationships between log-transformed dissolved P (mg P kg^-1^ soil) and plot-mean plant traits. Symbols depict the extraction method to estimate dissolved P: ALA (points) and Olsen (crosses). LNC and LPC were not examined because all plots for which LNC and LPC data was available (i.e. dataset 3) used Olsen extraction method. When the coefficients of both dissolved P and method of P extraction were significant (*p* <0.05) in multivariate regression, the regression lines are drawn for ALA (solid line) and for Olsen (broken line) separately.

**
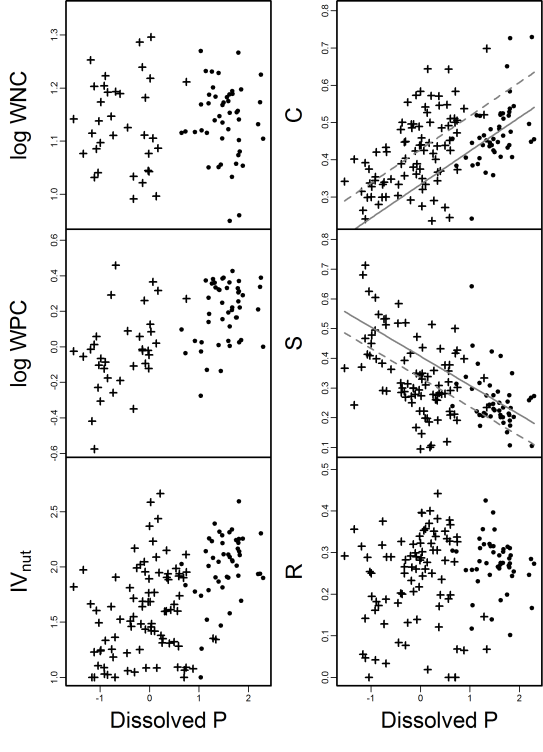
**

**Reference**

1. Carmo Horta Md, Roboredo M, Coutinho J, Torrent J (2010) Relationship between Olsen P and ammonium lactate-extractable P in Portuguese acid soils. Communications in Soil Science and Plant Analysis 41: 2358-2370.
