# Supplementary material for: Relationships between Nutrient-Related Plant Traits and Combinations of Soil N and P Fertility Measures
Source: PLoS One. 2013 Dec 31;8(12):e83735. doi: 10.1371/journal.pone.0083735 (PMC3877083; doi:10.1371/journal.pone.0083735)
Supplement: Appendix S2 — Testing the effects of using Olsen-P extraction for acid soils. (DOCX) [file pone.0083735.s005.docx]

**Appendix S2. Testing the effects of using Olsen-P extraction for acid soils**

Olsen-P extraction method is primarily meant for neutral to alkaline soils, although some previous studies used Olsen extractable P as an index of dissolved P availability for the purpose of comparing multiple sites with a wide range of pH [[e.g. 1](#_ENREF_1)]. A risk of using Olsen extraction for acid soils is that the amount of dissolved P could be overestimated for those soils, because aluminum and iron phosphates, if present, are dissolved as pH rises. Our dataset contains 41 plots (21 plots from dataset 1 and 20 plots from dataset 3) which were acid (pH_KCl_ < 5) and which used Olsen extraction method to estimate dissolved P. Dissolved P values for these sites are less reliable. In order to examine if these sites caused a bias in our findings, we tested the effects of dissolved P (relative to those of the other soil P measures) on plant traits by excluding these 41 plots.

Bivariate correlations between dissolved P and plant traits remained almost unchanged after excluding the 41 plots: Pearson’s correlation coefficients were 0.28 (n.s.) for LNC, 0.10 (n.s.) for LPC, 0.14 (n.s.) for WNC, 0.55 (*p*<0.001) for WPC, 0.45 (*p*<0.001) for IV_nut_, 0.36 (*p*<0.001) for C, -0.23 (*p*<0.05) for S, and -0.17 (n.s.) for R. For the C and S components, however, the independent effects of dissolved P became much smaller (see Fig. S2F and Fig. S2G compared to Fig. 2N and Fig. 2O respectively). Independent effects of dissolved P on these traits were significantly (*p*<0.05) larger than the other soil P measures in the main analysis (Table S3), but it was not the case when the 41 plots were excluded (*p*>0.05 with bootstrapping). Thus, the timescale effects of soil P measures on the C and S components observed in the main analysis could be an artifact of the measurement errors of dissolved P. For the other plant traits, the magnitudes of the independent effects of dissolved P remained almost unchanged. These results suggest that use of Olsen-P extraction method in acid soils did not have major consequences for our main findings except for the timescale effects of soil P measures for the C and S components.

**Figure S2.** Hierarchical partitioning of among-site plant trait variance into independent effects of a soil fertility measure (black bars) and its joint effects with other measures (white bars). Plots which were acid (pH<5) and which used Olsen extraction method (*n*=41) were excluded. Examined plot-mean plant traits are A: log LNC (mg/g) (*n=*31), B: log LPC (mg/g) (*n=*31), C: log WNC (mg/g) (*n=*62), D: log WPC (mg/g) (*n=*62), E: IV_nut_ (*n=*93), F: C component (*n=*93), G: S component (*n=*93), and H: R component (*n=*93). Stars indicate that the independent effect was significant based on Z-scores computed with randomization (^*^:*p* <0.05, ^**^: *p* <0.01, ^***^: *p* <0.001). 95% confidence intervals of independent effects, obtained by 1000-time bootstrapping, are shown.


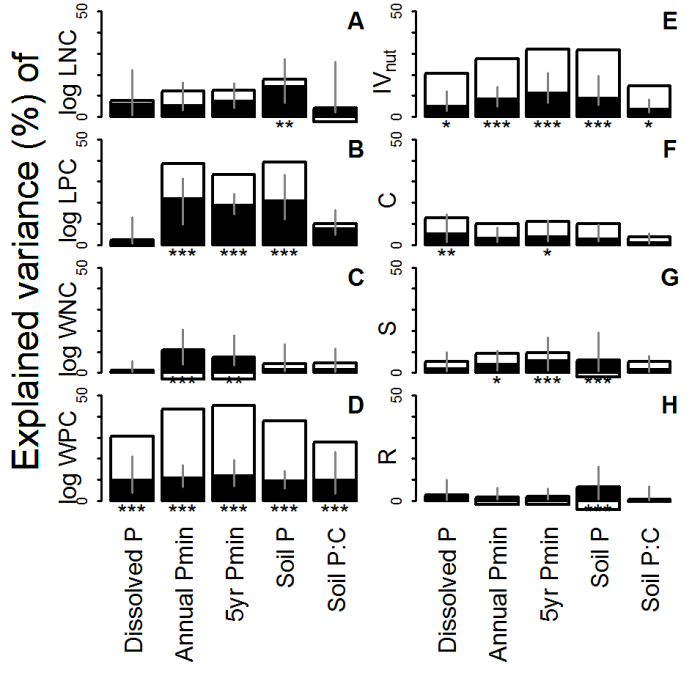


**Reference**

1. Ceulemans T, Merckx R, Hens M, Honnay O (2011) A trait-based analysis of the role of phosphorus vs. nitrogen enrichment in plant species loss across North-west European grasslands. Journal of Applied Ecology 48: 1155-1163.
