# Supplementary material for: Relationships between Nutrient-Related Plant Traits and Combinations of Soil N and P Fertility Measures
Source: PLoS One. 2013 Dec 31;8(12):e83735. doi: 10.1371/journal.pone.0083735 (PMC3877083; doi:10.1371/journal.pone.0083735)
Supplement: Appendix S3 — Fertility–trait relationships in N- and P-limited ecosystems. (DOCX) [file pone.0083735.s006.docx]

**Appendix S3. Fertility–trait relationships in N- and P-limited ecosystems**

In order to examine if our findings were not biased by the distribution of N- and P-limited plots of our dataset, we analyzed the relationships of soil fertility and plant traits separately for N- and P-limited plots. The type of limitation can be approximated by the N:P ratio of above-ground vascular plant biomass [[1](#_ENREF_1),[2](#_ENREF_2)]. For the sites which have no data on nutrient concentrations on a whole-canopy level (i.e. dataset 3: *n*=51), we used leaf-level nutrient concentrations to calculate plant N:P ratios. One site was excluded from the analysis because it did not have plant nutrient concentration data at all. With the threshold N:P ratio value of 15 [[2](#_ENREF_2)], our dataset was classified into 69 N-limited (N:P<15) and 64 P-limited (N:P>15) plots. The number of plots from each dataset was 16 (dataset 1), 43 (dataset 2), and 10 (dataset 3) for N-limited plots, and 19 (dataset 1), 4 (dataset 2), and 41 (dataset 3) for P-limited plots.

In addition, we tested the effect of the type of limitation on relationships between PCA axes (x) and plant traits (y), using a dummy variable *d* (1: N-limited, 0: P-limited) in multiple linear regression. The models tested were: *y* = *β*_0_ + *β*_1_·*x*, *y* = *β*_0_ + *β*_1_·*x* + *β*_2_·*d*, and *y* = *β*_0_ + *β*_1_·*x* + *β*_2_·*d* + *β*_3_·*x*·*d*. Among the models for which the coefficient of the PCA axis (*β_1_*) was significantly (*p* <0.05) different from zero, we chose the best model based on AIC.

When only N-limited plots were considered, contributions of soil N measures to plant trait variation tended to increase slightly (Fig. S3 left), but other major trends (e.g. stronger effect of soil P on WPC than that of soil N (*p* =0.094), small shared effects on CSR strategy) remained. When only P-limited plots were considered (Fig. S3 right), the contributions of soil P remained mostly unchanged, or even decreased for some traits. Here the major trends also remained. It is particularly notable that the stronger determinant for stress-tolerators were N-related fertility measures in N-limited plots (*p* <0.05) and P-related fertility measures in P-limited plots (*p* =0.079) (Fig. S3). Furthermore, signs of correlations between soil N measures and integrative plant traits (IV_nut_, CSR-strategy) reversed in P-limited plots compared to N-limited plots (e.g. stress-tolerator increased as soil N availability increased in P-limited plots only; see Table S4 in comparison to Table S5).

By including the type of nutrient limitation as a dummy variable, the effects of PCA axis 1 were modulated for WNC, WPC, IV_nut_, and R component, whereas those of PCA axis 2 were modulated for WPC, S component and R component (Fig. S4).

**Figure S3. Variation partitioning in N- and P-limited ecosystems**Variation partitioning (in percentage of variance) of among-site plant traits into unique effects of soil N measures (dark grey), unique effects of soil P measures (white), and shared effects of both (light grey) for N-limited (left) and P-limited (right) plots. Examined plant traits are A: log LNC (mg/g) (*n=*10 for N-limited and *n=*41 for P-limited), B: log WNC (mg/g) (*n=*10 and *n=*41), C: log LPC (mg/g) (*n=*59 and *n=*23), D: log WPC (mg/g) (*n=*59 and *n=*23), E: IV_nut_ (*n=*69 and *n=*64), F: C component (*n=*69 and *n=*64), G: S component (*n=*69 and *n=*64), and H: R component (*n=*69 and *n=*64). Results for LNC and LPC for N-limited ecosystems are not shown because they have too few samples (*n=*10). When contribution of N or P measures to total explained variance is significantly larger than the other, asterisks are given (^*^: *p*<0.05).


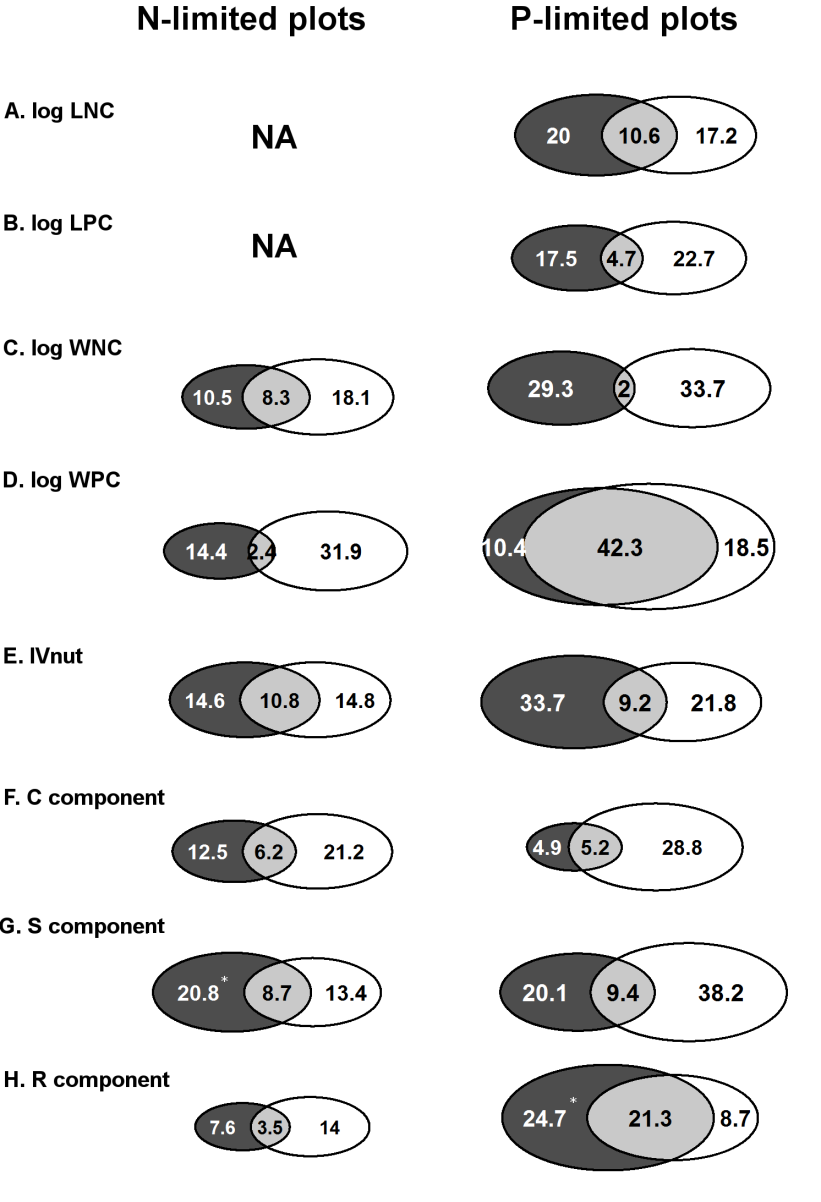


**Table S4.** Correlations between plot-mean plant traits and soil fertility measures in N-limited plots. Pearson’s correlation coefficient and *p*-values are shown. All variables were log-transformed prior to the analysis except for IV_nut_, C, S, and R.

|  | LNC | WNC | LPC | WPC | IV_nut_ | C | S | R |
| --- | --- | --- | --- | --- | --- | --- | --- | --- |
| Dissolved N | -0.58 ns | 0.19 ns | -0.05 ns | 0.29 * | 0.30 * | 0.37 ** | -0.36 ** | -0.02 ns |
| Summer Nmin | -0.28 ns | 0.05 ns | 0.01 ns | 0.33 * | 0.39 ** | 0.34 ** | -0.42 *** | 0.10 ns |
| Annual Nmin | -0.47 ns | 0.28 * | 0.04 ns | 0.18 ns | 0.20 ns | 0.07 ns | -0.14 ns | 0.07 ns |
| 5yr Nmin | -0.44 ns | 0.27 * | 0.02 ns | 0.20 ns | 0.22 ns | 0.09 ns | -0.15 ns | 0.06 ns |
| Soil N | -0.46 ns | 0.21 ns | 0.08 ns | 0.22 ns | 0.31 * | 0.15 ns | -0.24 * | 0.10 ns |
| Soil N:C | -0.04 ns | -0.09 ns | 0.49 ns | -0.24 ns | -0.05 ns | -0.31 ** | 0.19 ns | 0.14 ns |
| Dissolved P | -0.16 ns | 0.20 ns | -0.31 ns | 0.36 ** | 0.26 * | 0.42 *** | -0.33 ** | -0.11 ns |
| Annual Pmin | 0.42 ns | 0.47 *** | 0.68 * | 0.50 *** | 0.32 ** | 0.09 ns | -0.24 * | 0.17 ns |
| 5yr Pmin | 0.42 ns | 0.44 *** | 0.65 * | 0.50 *** | 0.33 ** | 0.07 ns | -0.22 ns | 0.16 ns |
| Soil P | 0.17 ns | 0.37 ** | 0.56 ns | 0.48 *** | 0.40 *** | 0.13 ns | -0.28 * | 0.18 ns |
| Soil P:C | 0.45 ns | 0.31 * | 0.69 * | 0.44 *** | 0.25 * | -0.10 ns | -0.09 ns | 0.22 ns |
| Soil N:P | -0.52 ns | -0.38 ** | -0.68 * | -0.58 *** | -0.30 * | -0.01 ns | 0.18 ns | -0.19 ns |
|  |  |  |  |  |  |  |  |  |
| N. of plots | 10 | 59 | 10 | 59 | 69 | 69 | 69 | 69 |

**Table S5.** Correlations between plot-mean plant traits and soil fertility measures in P-limited plots. Pearson’s correlation coefficient and *p*-values are shown. All variables were log-transformed prior to the analysis except for IV_nut_, C, S, and R.

|  | LNC | WNC | LPC | WPC | IVnut | C | S | R |
| --- | --- | --- | --- | --- | --- | --- | --- | --- |
| Dissolved N | 0.32 * | 0.10 ns | 0.01 ns | 0.22 ns | -0.03 ns | 0.21 ns | 0.11 ns | -0.31 * |
| Summer Nmin | 0.50 *** | 0.13 ns | 0.06 ns | 0.09 ns | -0.20 ns | -0.07 ns | 0.38 ** | -0.39 ** |
| Annual Nmin | 0.40 * | 0.13 ns | 0.10 ns | 0.11 ns | -0.11 ns | -0.12 ns | 0.38 ** | -0.34 ** |
| 5yr Nmin | 0.40 ** | 0.13 ns | 0.12 ns | 0.06 ns | -0.15 ns | -0.11 ns | 0.43 *** | -0.40 ** |
| Soil N | 0.42 ** | 0.03 ns | 0.20 ns | -0.08 ns | -0.12 ns | -0.11 ns | 0.44 *** | -0.41 *** |
| Soil N:C | 0.41 ** | 0.26 ns | 0.25 ns | 0.61 ** | 0.16 ns | -0.19 ns | 0.12 ns | 0.03 ns |
| Dissolved P | 0.37 * | 0.04 ns | 0.28 ns | 0.21 ns | 0.32 * | 0.41 *** | -0.44 *** | 0.15 ns |
| Annual Pmin | 0.22 ns | -0.02 ns | 0.13 ns | 0.52 * | 0.28 * | 0.34 ** | -0.23 ns | -0.03 ns |
| 5yr Pmin | 0.23 ns | 0.04 ns | 0.08 ns | 0.60 ** | 0.26 * | 0.32 ** | -0.20 ns | -0.06 ns |
| Soil P | 0.30 ns | 0.24 ns | 0.13 ns | 0.35 ns | -0.01 ns | 0.07 ns | 0.21 ns | -0.31 * |
| Soil P:C | 0.10 ns | 0.29 ns | 0.07 ns | 0.65 *** | 0.28 * | 0.15 ns | -0.33 ** | 0.25 * |
| Soil N:P | 0.16 ns | -0.26 ns | 0.09 ns | -0.56 ** | -0.17 ns | -0.25 * | 0.38 ** | -0.22 ns |
|  |  |  |  |  |  |  |  |  |
| N. of plots | 41 | 23 | 41 | 23 | 64 | 64 | 64 | 64 |

**Figure S4.** Relationships between PCA axes and plot-mean plant traits in N- and P-limited sites. Symbols depict the type of nutrient limitation of the site: N-limited (points) and P-limited (crosses). Among the regression models in which coefficient of the PCA axis was significantly different from zero, the best model was chosen (see text for details). When applicable, the regression lines are drawn for N-limited (solid line) and for P-limited (broken line) separately. Adjusted R^2^ and *p*-values are shown.


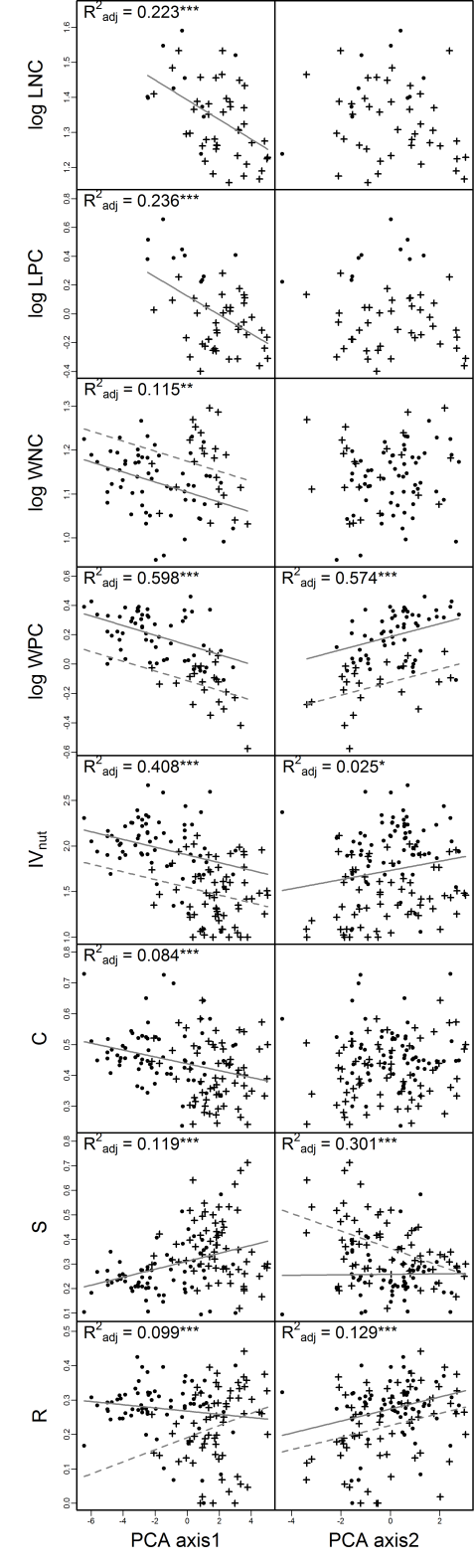


**Reference**

1. Olde Venterink H, Wassen MJ, Verkroost AWM, de Ruiter PC (2003) Species richness-productivity patterns differ between N-, P-, and K- limited wetlands. Ecology 84: 2191-2199.

2. Verhoeven JTA, Koerselman W, Meuleman AFM (1996) Nitrogen- or phosphorus-limited growth in herbaceous, wet vegetation: relations with atmospheric inputs and management regimes. Trends in Ecology and Evolution 11: 494-497.
