# Supplementary material for: Relationships between Nutrient-Related Plant Traits and Combinations of Soil N and P Fertility Measures
Source: PLoS One. 2013 Dec 31;8(12):e83735. doi: 10.1371/journal.pone.0083735 (PMC3877083; doi:10.1371/journal.pone.0083735)
Supplement: Appendix S4 — Testing additional soil P measures for a subset of dataset. (DOCX) [file pone.0083735.s007.docx]

**Appendix S4. Testing additional soil P measures for a subset of dataset**

With dataset 1 (*n*=36), for which three extra soil P measures were available, we tested whether these extra measures have stronger contributions to among-site variations of plant traits than the soil P measures examined in the main analysis, irrespective of the correlations among the soil P measures. The additional soil P measures included: summer gross P mineralization rates, ‘summer P min’, (mgP kg soil^-1^ 6weeks^-1^), roughly estimated as increase in Olsen-extractable labile P (including both organic and inorganic part) in in-situ soil incubations; oxalate extractable P, ‘Pox’ (mgP kg soil^-1^); and phosphorus saturation degree, ‘PSD’ (%), calculated as percentage of oxalate-extractable P over the half of oxalate-extractable Al and Fe [[1](#_ENREF_1)].
Only PSD had a significant independent effect on WPC (Fig. S5B, *p*<0.05), Moreover, none of the independent effects of the extra P fertility measures was larger than those of the other P fertility measures (*p*>0.05 with bootstrapping) (Fig. S5A-F). This indicates that the limited selection of soil P measures used in our main analysis did not bias our findings.

**Figure S5.** Hierarchical partitioning of among-site variance of plant traits into independent (black bars) and joint (white bars) effects of various P-related soil fertility measures. Plant traits examined are A: log WNC (mg/g) (*n=*35), B: log WPC (mg/g) (*n=*35), C: IV_nut_ (*n=*36), D: component (*n=*36), E: S component (*n=*36), and F: R component (*n=*36). Soil fertility measures were log-transformed prior to the analysis except for PSD. Asterisks indicate that the independent effect was significant based on Z-scores computed with randomization (^*^: *p* <0.05, ^**^: *p* <0.01, ^***^: *p* <0.001). 95% confidence intervals of independent effects, obtained by 1000-time bootstrapping, are shown.


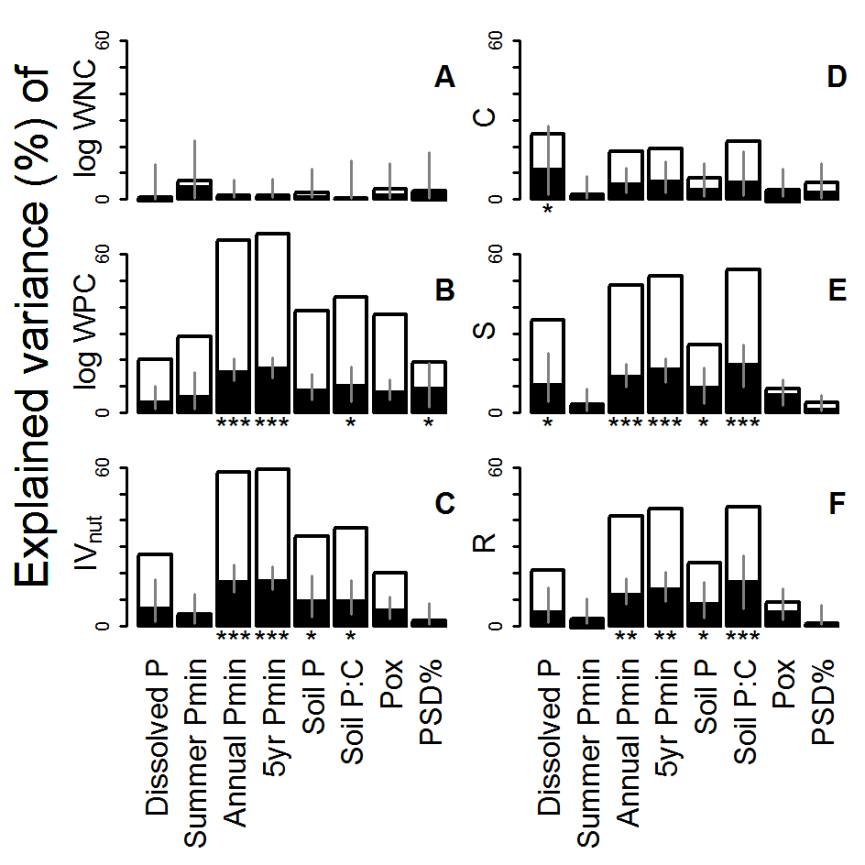


**Reference**

1. Lookman R, Vandeweert N, Merckx R, Vlassak K (1995) Geostatistical assessment of the regional distribution of phosphate sorption capacity parameters (FeOX and AlOX) in northern Belgium. Geoderma 66: 285-296.
